# Which dominates recurrence: tumor or microenvironment?

**DOI:** 10.1186/s12967-025-07193-9

**Published:** 2025-10-28

**Authors:** Guoqing Lyu, Xin Deng, Man Yang, Yan Guo, Yuan Zhang, Lihua Wang, Yan Huang, Sun Wu, Yanting Liu, Wenting Lv, Jincheng Guo

**Affiliations:** 1 Department of Hematology, The First Affiliated Hospital of Xinxiang Medical University, Weihui, Henan Province 453100 China; 2 Key Laboratory for Leukemia Molecular Diagnosis and Treatment in Xinxiang City, Weihui, Henan Province 453100 China; 3 Key Laboratory for Lymphoma Molecular Diagnosis and Treatment in Xinxiang City, Weihui, Henan Province 453100 China; 4 Cancer Research Institute, Cancer Hospital, The First Affiliated Hospital of Xinxiang Medical University, Weihui, Henan Province 453100 China; 5 School of Medicine, Pingdingshan University, Pingdingshan City, 467000 China; 6 Beijing University of Chinese Medicine, Beijing, 100029 China

**Keywords:** Lung squamous cell carcinoma, Recurrence, Single-cell RNA sequencing, Pathological response, Regulatory T cells

## Abstract

Lung squamous cell carcinoma (LUSC) is a major challenge in oncology due to its high recurrence rate, highlighting the need to identify recurrence drivers. Based on the data provided by Zhang Zemin’s team in their research, we selected survival metadata from 119 patients with lung squamous cell carcinoma and data on the proportions of 51 immune cell types. We then combined machine learning methods to analyze the intrinsic tumor factors and tumor microenvironment (TME) in the recurrence of lung squamous cell carcinoma (LUSC). Chi-square tests identified immune cell subsets enriched in recurrent patients, such as high-MKI67 Tregs, low-FGFBP2 NK cells, low-FOXP3 Tregs, and low-KLRB1 CD8 + T cells. A random forest algorithm further pinpointed pathological response rate (PRR) as the primary predictive factor, with high-MKI67 Tregs as a key secondary contributor. Univariate and multivariate Cox regression analyses and Kaplan-Meier survival curves confirmed that pathological complete response (pCR) and low Treg_MKI67 expression were associated with better survival (*p* = 0.0055 and *p* = 0.011, respectively). Patients achieving pCR and with low Treg_MKI67 expression had superior recurrence-free survival (RFS; 2-year RFS: 96.7% vs. 57.9% for non-pCR and high Treg_MKI67, *p* = 0.00066). These findings underscore the prognostic value of PRR and TME markers. They also highlight the potential of using single-cell RNA sequencing and machine learning to guide personalized therapies and reduce recurrence risk by targeting Treg activity, laying the groundwork for integrating immune markers into clinical practice to improve LUSC prognosis.

**Supplementary Information:**

The online version contains supplementary material available at 10.1186/s12967-025-07193-9.

Lung squamous cell carcinoma (LUSC) is a malignancy that remains a leading cause of cancer-related mortality worldwide [1]. Recurrence poses a significant barrier to improving patient outcomes, necessitating a deeper understanding of its underlying drivers. In this study, we leveraged single-cell RNA sequencing (scRNA-seq) and machine learning techniques to dissect the interplay between tumor-intrinsic characteristics and the tumor microenvironment (TME), identifying key predictors of recurrence-free survival (RFS). Our work builds on the foundational single-cell atlas from Zemin Zhang’s team, renowned for elucidating immune heterogeneity in anti-PD-1-treated non-small cell lung cancer (NSCLC) [2].

LUSC recurrence is influenced by complex interactions between tumor cells and their surrounding microenvironment, particularly leukocyte populations within the TME. These interactions can either promote tumor progression or facilitate regression, making the TME a critical focus for identifying prognostic biomarkers. To address this issue, we analyzed data from 119 patients with LUSC, which integrated clinical records, pathological assessments, and the proportion of each immune cell subtype in total CD45 + cells from single-cell RNA sequencing (scRNA-seq) data. Next, we integrated chi-square tests, random survival forests (RSF), univariate and multivariate Cox regression, and Kaplan-Meier survival analysis. We identified subpopulations significantly enriched in recurrent patients, which not only revealed the core factors driving the recurrence of lung squamous cell carcinoma but also further validated the prognostic value of PRR and TME biomarkers in this disease.

## Immune cell subsets associated with recurrence

The original paper pointed out that single-cell RNA sequencing (scRNA-seq) analysis identified 51 single-cell types significantly associated with lung squamous cell carcinoma (LUSC) recurrence (*p* < 0.05). Based on the proportion of these cell types and the relevant clinical survival metadata of patients, we analyzed the immune cell subpopulations that were significantly enriched in patients with recurrence. Distinct leukocyte populations within the tumor microenvironment emerged as pivotal determinants of recurrence: proliferative regulatory T cells (Tregs) with high MKI67 expression (Treg_MKI67_group High), natural killer (NK) cells with low FGFBP2 expression (NK_CD16hi_FGFBP2_group Low), Tregs with low FOXP3 expression (Treg_FOXP3_group Low), and CD8 + T cells with low KLRB1 expression (CD8T_MAIT_KLRB1_group Low) were all markedly enriched in patients who relapsed. These findings underscore the immune landscape’s decisive influence on LUSC outcomes, revealing how immunosuppressive subsets—particularly proliferative Tregs—may sculpt a microenvironment conducive to tumor relapse (Supplementary Table 1).

## Random survival forest analysis prioritizes predictors of RFS

**Fig. 1:**
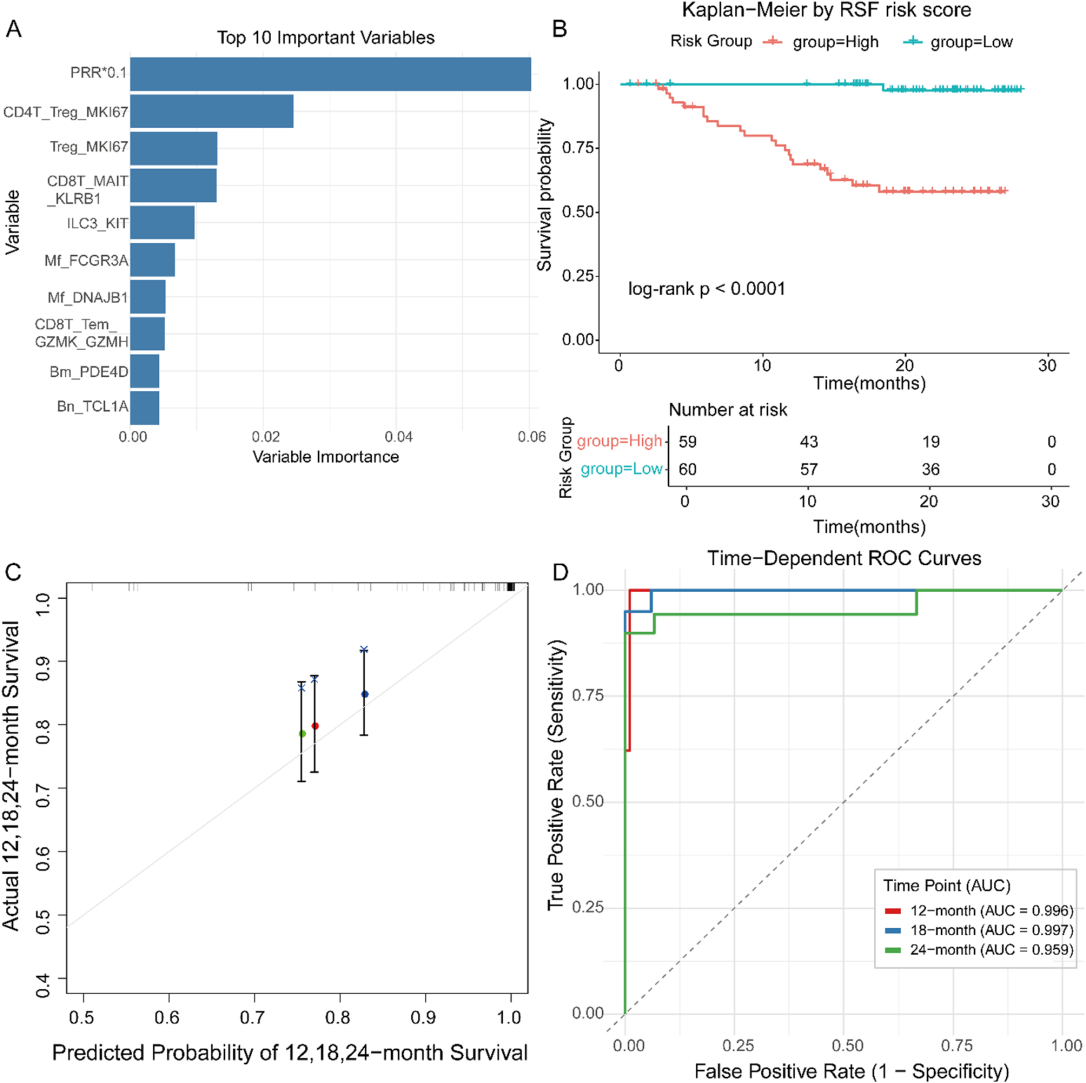
Random survival forest analysis prioritizes predictors of RFS. (**A**) Random forest screening ranks the top ten features by importance. (**B**) Patients are stratified into high- and low-risk groups by model-predicted survival scores, with significant differences in survival (*p* < 0.05). (**C**) Calibration curves for 12-, 18-, and 24-month survival predictions. (**D**) Area under the curve (AUC) values for 12-, 18-, and 24-month survival predictions are 0.996, 0.997, and 0.959, respectively

To quantify the contribution of each predictor to recurrence-free survival (RFS), we trained a Random Survival Forest (RSF) model, a non-parametric ensemble algorithm that ranks variables by their marginal impact on prediction accuracy. Importance scores were normalized, with the strongest contributor scaled to 1.0. PRR emerged as the single most influential determinant of RFS, attaining an importance score of 0.0605 (relative importance = 1.0000) (Fig. 1A). This dominant signal underscores the tumor’s therapeutic response as the cardinal arbiter of relapse risk. Immediately downstream, indices of regulatory T-cell proliferation—CD4T_Treg_MKI67 (0.0210; 0.3468) and Treg_MKI67 (0.0135; 0.2237)—rose to prominence, highlighting how tumor-intrinsic biology and immunosuppressive TME circuitry converge to shape LUSC recurrence. After stratifying patients into high- and low-risk groups using the constructed model, significant differences in survival were observed between the two groups (Fig. 1B). The calibration curve constructed to evaluate the predictive performance for patient survival at 12, 18, and 24 months showed good results (Fig. 1C). In addition, the model demonstrated excellent discriminatory ability, with AUC values of 0.996, 0.997, and 0.959 for 12-month, 18-month, and 24-month RFS, respectively (Fig. 1D).

## Cox regression confirms PRR and Treg effects

**Fig. 2:**
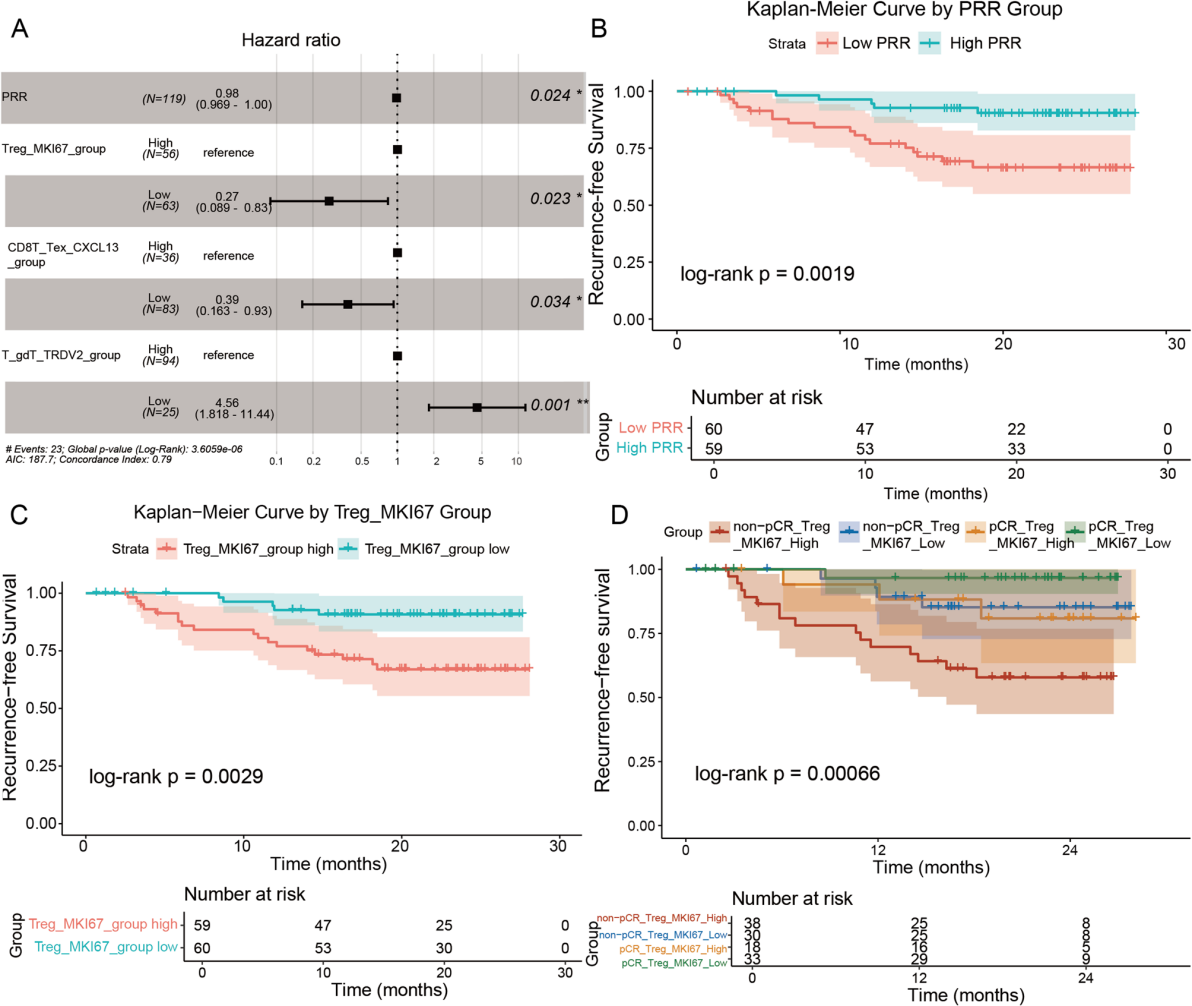
Statistical validation using Cox regression and survival analysis. (A) The results of the multivariate Cox analysis presented the hazard ratios (HR) for different cell types, including Treg_MKI67, CD8T_Tex_CXCL13, and T_gdT_TRDV2. Each cell type was divided into two groups: the high-expression group (High) and the low-expression group (Low), with the low-expression group serving as the reference group. (B) Kaplan-Meier curves for recurrence-free survival stratified by pathological response rate (PRR) demonstrated a statistically significant survival difference between the two groups (*P* < 0.05). (C) Kaplan-Meier curves for recurrence-free survival after stratification based on the proportion of regulatory T cells (Treg_MKI67) demonstrated a statistically significant survival difference between the two groups (*P* < 0.05). (D) Kaplan-Meier curves for recurrence-free survival based on further stratified analysis of the interaction between pCR (pathological complete response) status and regulatory T cell (Treg_MKI67) proportion, indicating statistically significant survival differences between groups (*P* < 0.05)

In univariate Cox regression analysis of patients, both PRR and Treg_MKI67 demonstrated significant associations with survival time. Specifically, increased PRR was significantly associated with shorter survival time (HR = 4.86, *P* < 0.001), indicating that PRR is a poor prognostic factor. Conversely, increased Treg_MKI67 was significantly associated with longer survival time (HR=0.225, *P* = 0.003), suggesting that Treg_MKI67 is a favorable prognostic factor (Supplementary Table 2). Further multivariable Cox regression analysis provided a more precise assessment of the effects of PRR and Treg_MKI67 after stratifying patients. In the multivariate analysis, increased PRR_High was significantly associated with prolonged survival time (HR = 0.969, 95% CI 0.941 – 0.998; *P* = 0.024), implying that each 1% increase in PRR_High independently reduced the risk of recurrence by 3.1%. This finding indicates that high PRR levels are associated with improved prognosis after accounting for the combined effects of other variables. Similarly, Treg_MKI67_Low also demonstrated significant association with survival time (HR = 0.27, 95% CI 0.09 – 0.81; *P* = 0.023), with low Treg proliferative activity (Treg_MKI67 Low) associated with a 73% risk reduction. This further underscores the importance of Treg proliferative activity in prognostic assessment, as low Treg_MKI67 levels may correlate with enhanced immune surveillance and improved outcomes (Fig. 2A).

Kaplan–Meier curves reinforced these findings: pathological complete response (pCR) was associated with markedly longer recurrence-free survival (*P* = 0.0019; Fig. 2B), and low Treg_MKI67 expression similarly predicted superior outcomes (*P* = 0.0029; Fig. 2C). Together, the data establish pathological response and restrained Treg proliferation as complementary, clinically actionable determinants of LUSC recurrence. 

## Combined pCR/Treg signature refines LUSC recurrence risk

Integrating pathological response and Treg activity sharpened prognostic resolution. When patients were cross-stratified by pCR status and Treg_MKI67 expression, Kaplan–Meier analysis revealed a striking gradient in recurrence-free survival (log-rank *P* = 0.00066). The most favorable cohort—pCR with low Treg_MKI67—achieved a 2-year RFS of 96.7% (95% CI 94.5–100%), whereas non-pCR patients burdened by high Treg_MKI67 fared worst at 57.9% (95% CI 43.6–76.8%). Among non-pCR cases, low Treg_MKI67 elevated 2-year RFS to 85.2% (95% CI 72.8–99.8%), numerically surpassing even pCR patients with high Treg_MKI67 (80.9%, 95% CI 63.4–100%), although this difference lacked statistical significance (Fig. 2D). Thus, restrained Treg proliferation amplifies the benefit of pathological response and mitigates the penalty of residual disease, underscoring the value of combined pathological and immune profiling for refined risk stratification in LUSC.

## Implications for LUSC management

Our findings weave tumor-intrinsic biology and microenvironmental circuitry into a unified map of LUSC recurrence. The commanding influence of PRR sets a clear therapeutic imperative: a robust pathological response is not merely an endpoint, but a gateway to durable cure. For instance, the CheckMate 816 trial demonstrated that neoadjuvant nivolumab plus chemotherapy significantly improved event-free survival (EFS) and pCR in resectable lung cancer, with pCR and major pathological response (MPR, defined as ≤ 10% viable tumor) associated with better outcomes [3]. Similarly, studies have validated MPR as a surrogate endpoint for overall survival (OS), supporting our observation that higher PRR reduces recurrence risk (*p* < 0.001) [4]. PRR thus emerges as a ready biomarker to triage patients toward—or away from—adjuvant intensification.

Yet therapeutic success is not sealed by tumor regression alone. The conspicuous footprint of proliferative Tregs reveals an immunosuppressive TME poised to sabotage even the most impressive pathological responses. Single-cell atlases and spatial transcriptomics now converge on this theme: CCR4⁺ effector Tregs portend poor relapse-free survival when they exceed 40% of the total Treg pool (OR 6.54, *p* = 0.007), while spatial mapping of Treg neighborhoods independently forecasts recurrence in resectable NSCLC [5]. Our data align precisely—Treg_MKI67-high signatures amplify risk—suggesting that proliferative Tregs erect molecular barricades within the TME that chemoradiation alone cannot dismantle. Additionally, recent work on spatial cell interplay networks of Tregs predicting recurrence in operable NSCLC highlights the importance of Treg spatial distribution, complementing the scRNA-seq approach [6]. Compared with previous gene/methylation studies, we have supplemented our research with a multi-modal perspective at the cellular level [7–9]. At the same time, discoveries at the gene level provide indispensable molecular clues for elucidating the driving mechanisms. The two complement each other, laying a joint framework for subsequent pathological verification and personalized follow-up/treatment strategies.

Crucially, the protective gradient we observe with low Treg proliferation offers a therapeutic lever. Selective depletion or functional re-programming of Tregs—whether via CCR4 antagonists, low-dose IL-2 variants, or combination regimens—could convert residual disease into immunologically vulnerable targets. Single-cell resolution not only pinpoints these cellular liabilities but also quantifies their prognostic weight through RSF, yielding a blueprint for precision adjuvant trials where PRR and Treg burden jointly guide the intensity and modality of post-operative therapy.

## Implications for therapy and future directions

Leveraging patient survival metadata and immune-cell proportions, we constructed a prognostic model that enables precise risk stratification for lung squamous-cell carcinoma recurrence. Integrating pathological response rate (PRR) with immune-microenvironment markers—especially Treg_MKI67—not only surpasses traditional staging in accuracy but also slots readily into routine pathology workflows. Elevated Treg proliferation defines high-risk residual disease in non-pathological complete responders (non-pCR); thus, agents that curb Treg expansion could potentiate anti-tumor immunity in this vulnerable population. The recent conditional approval of ivonescimab for advanced NSCLC in China exemplifies how bispecific checkpoint blockade can overcome monotherapy resistance. Combining such antibodies with Treg-directed drugs—low-dose IL-2 variants, CCR4 antagonists, or PI3Kδ inhibitors—may tilt the balance toward durable tumor control [10]. Prospective validation in larger, multi-institutional cohorts, coupled with functional dissection of Treg heterogeneity and rational design of tumor-plus-microenvironment combination regimens, now constitute the critical next steps.

## Conclusion

This study establishes pathological response and regulatory T-cell activity as the twin axes governing recurrence-free survival in LUSC. By fusing single-cell resolution with machine-learning prioritization, we have mapped the immune topography that seeds relapse and extracted biomarkers that may be applied in future clinical applications. These insights lay the groundwork for precision adjuvant strategies that match tumor biology with microenvironment vulnerabilities, offering a clear path to improved outcomes for patients with LUSC. 

## Supplementary Information

Below is the link to the electronic supplementary material.

Supplementary Material 1

Supplementary Material 2

## Data Availability

Data for this study were obtained from publicly available datasets provided by the Zemin Zhang team.
