# Supplementary material for: Which dominates recurrence: tumor or microenvironment?
Source: J Transl Med. 2025 Oct 28;23:1181. doi: 10.1186/s12967-025-07193-9 (PMC12570626; doi:10.1186/s12967-025-07193-9)
Supplement: Supplementary file 1 — Supplementary Material 1 [file 12967_2025_7193_MOESM1_ESM.docx]

Supplementary table 1. Clinical Characteristics

| **Characteristic** | **N** | **RFS_status No**  N = 96^1^ | **RFS_status Yes**  N = 23^1^ | **p-value**^2^ |
| --- | --- | --- | --- | --- |
| PRR | 119 | 100.00 (80.00, 100.00) | 50.00 (30.00, 95.50) | <0.001 |
| RFS_months | 119 | 21 (17, 25) | 11 (6, 14) | <0.001 |
| B_prf_MKI67_group | 119 |  |  | 0.8 |
| High |  | 47 (49%) | 12 (52%) |  |
| Low |  | 49 (51%) | 11 (48%) |  |
| Bm_CD74_group | 119 |  |  | 0.5 |
| High |  | 46 (48%) | 13 (57%) |  |
| Low |  | 50 (52%) | 10 (43%) |  |
| Bm_FCRL4_group | 119 |  |  | 0.5 |
| High |  | 46 (48%) | 13 (57%) |  |
| Low |  | 50 (52%) | 10 (43%) |  |
| Bm_MT2A_group | 119 |  |  | 0.5 |
| High |  | 49 (51%) | 10 (43%) |  |
| Low |  | 47 (49%) | 13 (57%) |  |
| Bm_PDE4D_group | 119 |  |  | 0.5 |
| High |  | 49 (51%) | 10 (43%) |  |
| Low |  | 47 (49%) | 13 (57%) |  |
| Bm_TNF_group | 119 |  |  | 0.8 |
| High |  | 47 (49%) | 12 (52%) |  |
| Low |  | 49 (51%) | 11 (48%) |  |
| Bm_TNFSF9_group | 119 |  |  | 0.8 |
| High |  | 47 (49%) | 12 (52%) |  |
| Low |  | 49 (51%) | 11 (48%) |  |
| Bn_TCL1A_group | 119 |  |  | 0.095 |
| High |  | 44 (46%) | 15 (65%) |  |
| Low |  | 52 (54%) | 8 (35%) |  |
| CD4T_Tem_GZMA_group | 119 |  |  | 0.11 |
| High |  | 51 (53%) | 8 (35%) |  |
| Low |  | 45 (47%) | 15 (65%) |  |
| CD4T_Tfh_CXCL13_group | 119 |  |  | 0.8 |
| High |  | 47 (49%) | 12 (52%) |  |
| Low |  | 49 (51%) | 11 (48%) |  |
| CD4T_Th1_like_CXCL13_group | 119 |  |  | 0.5 |
| High |  | 46 (48%) | 13 (57%) |  |
| Low |  | 50 (52%) | 10 (43%) |  |
| CD4T_Tm_ANXA1_group | 119 |  |  | 0.3 |
| High |  | 50 (52%) | 9 (39%) |  |
| Low |  | 46 (48%) | 14 (61%) |  |
| CD4T_Tm_XCL1_group | 119 |  |  | 0.3 |
| High |  | 50 (52%) | 9 (39%) |  |
| Low |  | 46 (48%) | 14 (61%) |  |
| CD4T_Tn_CCR7_group | 119 |  |  | 0.8 |
| High |  | 47 (49%) | 12 (52%) |  |
| Low |  | 49 (51%) | 11 (48%) |  |
| CD4T_Treg_CCR8_group | 119 |  |  | 0.8 |
| High |  | 47 (49%) | 12 (52%) |  |
| Low |  | 49 (51%) | 11 (48%) |  |
| CD4T_Treg_FOXP3_group | 119 |  |  | 0.8 |
| High |  | 47 (49%) | 12 (52%) |  |
| Low |  | 49 (51%) | 11 (48%) |  |
| CD4T_Treg_MKI67_group | 119 |  |  | 0.2 |
| High |  | 45 (47%) | 14 (61%) |  |
| Low |  | 51 (53%) | 9 (39%) |  |
| CD8T_ISG15_group | 119 |  |  | 0.9 |
| High |  | 48 (50%) | 11 (48%) |  |
| Low |  | 48 (50%) | 12 (52%) |  |
| CD8T_MAIT_KLRB1_group | 119 |  |  | 0.012 |
| High |  | 53 (55%) | 6 (26%) |  |
| Low |  | 43 (45%) | 17 (74%) |  |
| CD8T_NK_like_FGFBP2_group | 119 |  |  | 0.11 |
| High |  | 51 (53%) | 8 (35%) |  |
| Low |  | 45 (47%) | 15 (65%) |  |
| CD8T_prf_MKI67_group | 119 |  |  | 0.5 |
| High |  | 49 (51%) | 10 (43%) |  |
| Low |  | 47 (49%) | 13 (57%) |  |
| CD8T_Tem_GZMK_GZMH_group | 119 |  |  | 0.3 |
| High |  | 50 (52%) | 9 (39%) |  |
| Low |  | 46 (48%) | 14 (61%) |  |
| CD8T_Tem_GZMK_NR4A1_group | 119 |  |  | 0.5 |
| High |  | 49 (51%) | 10 (43%) |  |
| Low |  | 47 (49%) | 13 (57%) |  |
| CD8T_terminal_Tex_LAYN_group | 119 |  |  | 0.5 |
| High |  | 46 (48%) | 13 (57%) |  |
| Low |  | 50 (52%) | 10 (43%) |  |
| CD8T_Tex_CXCL13_group | 119 |  |  | 0.5 |
| High |  | 46 (48%) | 13 (57%) |  |
| Low |  | 50 (52%) | 10 (43%) |  |
| CD8T_Tm_IL7R_group | 119 |  |  | 0.8 |
| High |  | 47 (49%) | 12 (52%) |  |
| Low |  | 49 (51%) | 11 (48%) |  |
| CD8T_Trm_ZNF683_group | 119 |  |  | 0.9 |
| High |  | 48 (50%) | 11 (48%) |  |
| Low |  | 48 (50%) | 12 (52%) |  |
| cDC1_CLEC9A_group | 119 |  |  | 0.5 |
| High |  | 46 (48%) | 13 (57%) |  |
| Low |  | 50 (52%) | 10 (43%) |  |
| cDC2_CD1C_group | 119 |  |  | 0.5 |
| High |  | 49 (51%) | 10 (43%) |  |
| Low |  | 47 (49%) | 13 (57%) |  |
| GCB_RGS13_group | 119 |  |  | 0.2 |
| High |  | 45 (47%) | 14 (61%) |  |
| Low |  | 51 (53%) | 9 (39%) |  |
| ILC3_KIT_group | 119 |  |  | 0.3 |
| High |  | 50 (52%) | 9 (39%) |  |
| Low |  | 46 (48%) | 14 (61%) |  |
| Mast_cell_group | 119 |  |  | 0.3 |
| High |  | 50 (52%) | 9 (39%) |  |
| Low |  | 46 (48%) | 14 (61%) |  |
| mDC_LAMP3_group | 119 |  |  | 0.9 |
| High |  | 48 (50%) | 11 (48%) |  |
| Low |  | 48 (50%) | 12 (52%) |  |
| Mφ_CXCL10_group | 119 |  |  | 0.5 |
| High |  | 46 (48%) | 13 (57%) |  |
| Low |  | 50 (52%) | 10 (43%) |  |
| Mφ_CXCL2_group | 119 |  |  | 0.8 |
| High |  | 47 (49%) | 12 (52%) |  |
| Low |  | 49 (51%) | 11 (48%) |  |
| Mφ_DNAJB1_group | 119 |  |  | 0.8 |
| High |  | 47 (49%) | 12 (52%) |  |
| Low |  | 49 (51%) | 11 (48%) |  |
| Mφ_FCGR3A_group | 119 |  |  | 0.3 |
| High |  | 50 (52%) | 9 (39%) |  |
| Low |  | 46 (48%) | 14 (61%) |  |
| Mφ_FOLR2_group | 119 |  |  | 0.8 |
| High |  | 47 (49%) | 12 (52%) |  |
| Low |  | 49 (51%) | 11 (48%) |  |
| Mφ_ISG15_group | 119 |  |  | 0.8 |
| High |  | 47 (49%) | 12 (52%) |  |
| Low |  | 49 (51%) | 11 (48%) |  |
| Mφ_MARCO_group | 119 |  |  | 0.8 |
| High |  | 47 (49%) | 12 (52%) |  |
| Low |  | 49 (51%) | 11 (48%) |  |
| Mφ_MKI67_group | 119 |  |  | 0.5 |
| High |  | 46 (48%) | 13 (57%) |  |
| Low |  | 50 (52%) | 10 (43%) |  |
| Mφ_MMP9_group | 119 |  |  | 0.5 |
| High |  | 49 (51%) | 10 (43%) |  |
| Low |  | 47 (49%) | 13 (57%) |  |
| Mφ_S100A10_group | 119 |  |  | 0.5 |
| High |  | 49 (51%) | 10 (43%) |  |
| Low |  | 47 (49%) | 13 (57%) |  |
| Mφ_VCAN_group | 119 |  |  | 0.5 |
| High |  | 49 (51%) | 10 (43%) |  |
| Low |  | 47 (49%) | 13 (57%) |  |
| Neu_FCGR3B_group | 119 |  |  | 0.8 |
| High |  | 47 (49%) | 12 (52%) |  |
| Low |  | 49 (51%) | 11 (48%) |  |
| NK_CD16hi_FGFBP2_group | 119 |  |  | 0.041 |
| High |  | 52 (54%) | 7 (30%) |  |
| Low |  | 44 (46%) | 16 (70%) |  |
| NK_CD16low_GZMK_group | 119 |  |  | 0.3 |
| High |  | 50 (52%) | 9 (39%) |  |
| Low |  | 46 (48%) | 14 (61%) |  |
| pDC_LILRA4_group | 119 |  |  | 0.5 |
| High |  | 46 (48%) | 13 (57%) |  |
| Low |  | 50 (52%) | 10 (43%) |  |
| Plasma_cell_group | 119 |  |  | 0.9 |
| High |  | 48 (50%) | 11 (48%) |  |
| Low |  | 48 (50%) | 12 (52%) |  |
| T_gdT_TRDV1_group | 119 |  |  | 0.5 |
| High |  | 49 (51%) | 10 (43%) |  |
| Low |  | 47 (49%) | 13 (57%) |  |
| T_gdT_TRDV2_group | 119 |  |  | 0.9 |
| High |  | 48 (50%) | 11 (48%) |  |
| Low |  | 48 (50%) | 12 (52%) |  |
| Treg_CCR8*10_group | 119 |  |  | 0.2 |
| High |  | 45 (47%) | 14 (61%) |  |
| Low |  | 51 (53%) | 9 (39%) |  |
| Treg_FOXP3_group | 119 |  |  | 0.041 |
| High |  | 52 (54%) | 7 (30%) |  |
| Low |  | 44 (46%) | 16 (70%) |  |
| Treg_MKI67_group | 119 |  |  | 0.002 |
| High |  | 41 (43%) | 18 (78%) |  |
| Low |  | 55 (57%) | 5 (22%) |  |
| RFS time | 119 | 21 (17, 25) | 11 (6, 14) | <0.001 |
| ^1^Median (Q1, Q3); n (%) | | | | |
| ^2^Wilcoxon rank sum test; Pearson's Chi-squared test; Fisher's exact test | | | | |
